# Leading and managing a team

**Published:** 2018-07-31

**Authors:** Dan Kiage

**Affiliations:** 1 Medical Director: Kisii Eye Hospital, Kisii, Kenya.

**Team leaders can ensure that an eye clinic or programme is effective by encouraging and maintaining the morale, motivation and efficiency of the team.**

**Figure:**
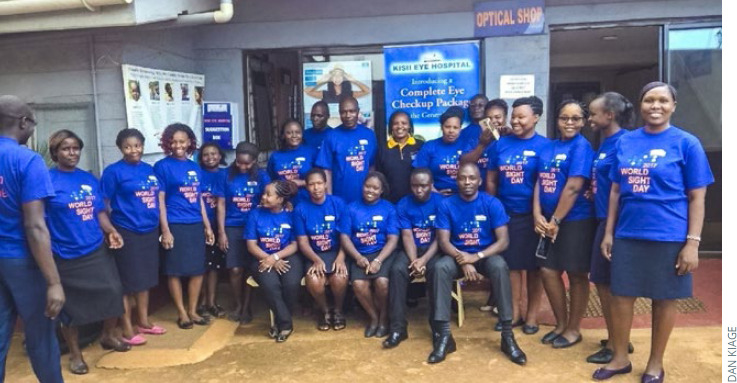
The team at Kisii Eye Hospital. KENYA

As mentioned in the article by Daksha Patel and Suzanne Gilbert (p. 48–50), it is widely acknowledged that a team approach improves the ability of eye hospitals, clinics and programmes to be effective and achieve their goals. This can only occur when team members are well organised, determined, motivated and efficient.

When setting up and managing a new eye hospital in Kenya, I quickly learnt that the greatest responsibility for shaping an effective team belongs to the team leader.

**“Leaders have to create and maintain the values and culture of the organisation (its ‘DNA’) and be champions of its goals.”**

## Be a leader

Team leaders have to possess skill and energy and must be well motivated and visionary. They have to be good role models, good listeners and fair in all that they do. Leaders have to create and maintain the values and culture of the organisation (its ‘DNA’) and be champions of its goals. They must actively motivate team members to be excited about the progress and direction of the organisation.

## Appoint team members to positions that suit them

The team leader has to hire and place individuals in appropriate positions that suit them best, which creates a great starting position for the team as a whole. For instance, a quiet, serious nurse may be a good fit in surgical theatre, whereas nurse who is a talkative ‘people person’ will be better placed in the clinic. This can be reviewed over time and changes made as leaders come to understand everyone's strengths.

## Set rules and boundaries

A staff manual is necessary to enable the workers to know the dos and don'ts in the organisation. The manual must provide guidelines about reporting structures and methods of conflict resolution.

## Support effective communication

Set up clear management structures that show how the senior management team (SMT) and heads of departments work together and communicate these to the whole team. The SMT must have frequent meetings, chaired by the team leader, to set the pace and direction of the group. Departments must have their own meetings and encourage everyone in the department to share their views. Depending on the size of the organisation, it is advisable for the whole team to have frequent meetings to remind them of the organisation's shared vision.

## Create good working environment

To ensure optimum productivity, set up the workplace as somewhere team members will be happy to spend their time. For example, ensure there are appropriate toilets and good temperature regulation, as well as lunch arrangements and tea/coffee breaks. Create a good atmosphere so people can bond as a team – an appropriate sense of humour is vital! The team should be proud to work at the hospital.

***Community Eye Health Journal* Data protection**

**Figure:**
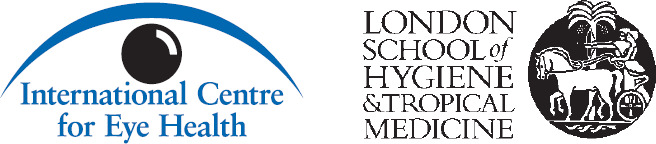

The United Kingdom's data protection laws are changing to protect customers and members of the public. The new General Data Protection Regulation (GDPR) sets out strict guidelines for the way we gather and protect your data.

Because you have subscribed to the *Community Eye Health Journal* (the *Journal*), the law permits us to stay in touch with you and to send you new issues of the *Journal* via email newsletter and/or by post. The law also gives you the right to unsubscribe from our email newsletter and to cancel your paper subscription at any time.

The *Journal* is published by the London School of Hygiene and Tropical Medicine (LSHTM). For more information, please consult the LSHTM **data protection policy**, which also explains the School's approach to privacy and security. Information about how this applies to the Community Eye Health Journal is available on **www.cehjournal.org/privacy-policy**.

If you wish to cancel your subscription to the Journal and unsubscribe from our communication list, please email **admin@cehjournal.org**. We will remove you from our mailing list within a reasonable time.

